# Genome-Wide Identification and Expression Pattern Analysis of BAHD Acyltransferase Family in *Taxus mairei*

**DOI:** 10.3390/ijms25073777

**Published:** 2024-03-28

**Authors:** Donghuan Xu, Zhong Wang, Weibing Zhuang, Fan Zhang, Yinfeng Xie, Tao Wang

**Affiliations:** 1 Jiangsu Key Laboratory for the Research and Utilization of Plant Resources, Institute of Botany, Jiangsu Province and Chinese Academy of Sciences (Nanjing Botanical Garden Mem. Sun Yat-Sen), Nanjing 210014, China; xu15689774892@163.com (D.X.); wangzhong@cnbg.net (Z.W.); weibingzhuang@cnbg.net (W.Z.);; 2 Co-Innovation Center for Sustainable Forestry in Southern China, College of Life Sciences, Nanjing Forestry University, Nanjing 210037, China; xxyyff@njfu.edu.cn

**Keywords:** genome-wide identification, TwBAHD acyltransferases, *Taxus mairei*, expression pattern, taxol biosynthesis

## Abstract

BAHD acyltransferases are involved in catalyzing and regulating the secondary metabolism in plants. Despite this, the members of BAHD family and their functions have not been reported in the *Taxus* species. In this study, a total of 123 TwBAHD acyltransferases from *Taxus wallichiana* var. *mairei* genome were identified and divided into six clades based on phylogenetic analysis, of which Clade VI contained a *Taxus*-specific branch of 52 members potentially involved in taxol biosynthesis. Most TwBAHDs from the same clade shared similar conserved motifs and gene structures. Besides the typical conserved motifs within the BAHD family, the YPLAGR motif was also conserved in multiple clades of *T. mairei*. Moreover, only one pair of tandem duplicate genes was found on chromosome 1, with a Ka/Ks ratio < 1, indicating that the function of duplicate genes did not differentiate significantly. RNA-seq analysis revealed different expression patterns of *TwBAHDs* in MeJA induction and tissue-specific expression experiments. Several *TwBAHD* genes in the *Taxus*-specific branch were highly expressed in different tissues of *T. mairei*, suggesting an important role in the taxol pathway. This study provides comprehensive information for the TwBAHD gene family and sets up a basis for its potential functions.

## 1. Introduction

*Taxus* (known as yews) are evergreen coniferous shrubs or trees in the family Taxaceae, and are rare and endangered gymnosperm species [1]. *Taxus* has served as a natural source of paclitaxel (trade name taxol), a tetracyclic diterpenoid compound, which first gained marketing approval from the U.S. Food and Drug Administration (FDA) for the clinical treatment of leukemia, Kaposi’s sarcoma, lung, breast, ovarian, and non-small-cell lung cancers [2,3,4]. However, plant-derived taxol is far from meeting the growing market demand due to the extremely low abundance in *Taxus*. For the foreseeable future, multiple strategies and promising progress for taxol supply issues will depend directly or indirectly on the elucidation of *Taxus* biosynthesis [5].

The taxol biosynthesis consists of 19 enzymatic steps, which could be divided into three stages: the formation and modification of the core taxane skeleton, the synthesis of *β*-phenylalanyl-CoA side chain, and the assembly of taxol [6]. Among them, the acylation reactions in the taxol pathway mediated by a series of specialized taxane acyltransferases, including taxadiene-5α-ol-O-acetyl transferase (TAT), taxane-2α-O-benzoyl transferase (TBT), 10-deacetylbaccatin III-10-O-acetyl transferase (DBAT), baccatin III: 3-amino, 3-phenylpropanoyl transferase (BAPT), and N-benzoyl transferase (DBTNBT) [7,8,9], are crucial for the yield of taxol. For example, TAT catalyzes the conversion of taxadien-5α-ol to taxadien-5α-yl acetate, which is a slow step for the downstream hydroxylation reactions [10,11,12]. The enzyme DBAT, catalyzing the acetylation of 10-deacetyl baccatin III (10-DAB) to yield baccatin III, is considered a key rate-limiting enzyme of the taxol pathway [13]. Notably, these taxane acyltransferases were all from the BAHD acyltransferase family and form a *Taxus*-specific branch [14]. However, the limited genetic information of *Taxus* has limited research on the origin, characteristics, and phylogeny for these acyltransferases.

The BAHD acyltransferase family is a group of proteins that use acyl-coenzyme A as the acyl donor and catalyze a series of oxygenated and nitrogenous compounds to produce various volatile lipids, modified anthocyanins, and compounds related to plant resistance to pathogenic microorganisms in plants [15]. It was named according to the first letter of each of the first four biochemically characterized enzymes within this family: benzylalcohol O-acetyltransferase (BEAT), anthocyanin O-hydroxycinnamoyltransferase (AHCT), anthranilate N-hydroxycinnamoyl/benzoyltransferase (HCBT), and deacetylvindoline 4-O-acetyltransferase (DAT) [16,17,18,19]. Members in this family share two conserved motifs: HXXXD and DFGWG [20]. The HXXXD motif is located near the center of each enzyme, which is essential for catalysis, while the DFGWG motif is located near the C-terminal of the protein and plays an important role in the binding of CoA [21]. Moreover, the BAHDs responsible for the synthesis of anthocyanins often contain an additional conserved motif, YFGNC [22].

In our previous study, the BAHD family was divided into six clades based on the type of substrates or the activity conditions of the enzymes [23]. The members of Clade I are mostly involved in the acylation of flavonoids, anthocyanins, and phenolic glucosides. Clade II is involved in the elongation of epicuticular waxes for preventing tissue moisture loss and resisting pathogen attack [24]. Clade III contains a series of alcohol acyltransferases involved in the biosynthesis of volatile lipids in flowers and mature fruits. Clade IV shares agmatine coumaroyltransferases (ACTs), whose DFGWG motif is slightly altered with glycine instead of tryptophan [25,26,27,28]. Clade V mainly contains members with hydroxycinnamoyl transferase (HCT) activity [29]. Clade VI shows diverse activities which utilize substrates ranging from terpenoids to medium-chain alcohols. Specialized taxane acyltransferases were in association with major phylogenetic branches within this clade. However, studies on BAHD acyltransferases of *Taxus* have not been further described.

In the current study, a comprehensive investigation of *Taxus wallichiana* var. *mairei* BAHD (TwBAHD) acyltransferases was conducted, and the sequence features, analysis of phylogenetic relationships, motif recognition, identification of exon/intron structures and cis-acting promoter elements, chromosome distribution, and gene replication were performed based on the whole genome of *T. mairei* [30]. Moreover, for identified TwBAHD genes, an induction expression analysis in *T. mairei* cells as well as a tissue-specific expression analysis in *T. mairei* trees using previous RNA-seq data were also performed. The expression levels of representative TwBAHD genes within the species-specific clade were also evaluated by qRT-PCR analysis. These findings should not only provide a characterization of the TwBAHD family, but also provide valuable information for further functional elucidation of these members in *Taxus*, which laid a foundation for the protection of *Taxus* resources and the mass production of taxol through genetic engineering.

## 2. Results

### 2.1. Identification of TwBAHD Family

To identify putative TwBAHD members, HMMER was performed to search the BAHD protein in the genome of *T. mairei*, and 124 putative protein sequences were obtained. Then, the SMART database and NCBI conservative domain database were applied to verify the conserved domains. A total of 123 TwBAHD protein sequences were finally retained, and were designated as TwBAHD1 to TwBAHD123 after deleting redundant sequences. The gene ID and protein ID for each sequence derived from *T. mairei* are provided in Table S2. The basic physical and chemical properties of the TwBAHD proteins such as amino acid number, molecular weight (MW), isoelectric point (pI), instability index, hydrophobicity prediction, and subcellular localizations are summarized in Table S3. In this study, the TwBAHD proteins ranged from 53 to 576 amino acids, with molecular weights between 5.87 and 63.85 kDa, and pI values between 5.30 and 10.00. The GRAVY of TwBAHD proteins ranged from −0.666 to 0.319, with 54% (67/123) being lower than zero, indicating that half of the proteins in the family were hydrophilic. Moreover, 59% (73/123) of TwBAHDs exhibited an instability index higher than 40, and no protein had a signal peptide. The subcellular location predicted result showed that 62 *TwBAHD* genes could be located in the cytoplasm, 32 were located in the chloroplast, 15 in nucleus, 7 in peroxisome, 3 in mitochondrion, 2 in cytoplasm-nucleus, 1 in cytoskeleton, and 1 in extracellular.

### 2.2. Phylogenetic Analysis of TwBAHD Family

**Figure 1:**
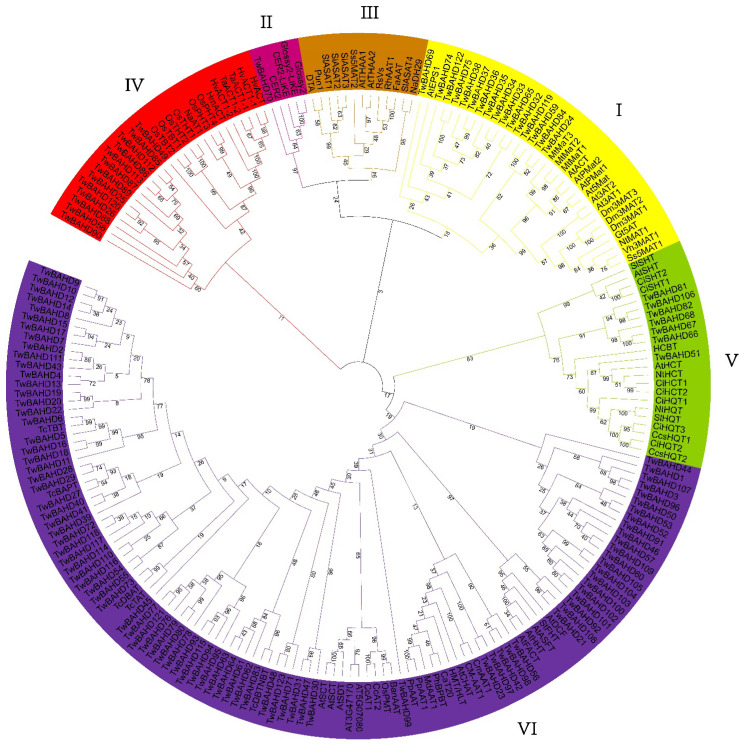
Phylogenetic tree of 123 BAHD proteins in *T. mairei* and 89 representative BAHD proteins in 31 species. An un-rooted phylogenetic tree of BAHD gene family among *T. mairei* and 31 other species was constructed using the maximum likelihood method with a bootstrap test (replicated 1000 times). Different colors represent different clades.

To investigate the evolutionary relationships of the TwBAHDs to those from other species, a phylogenetic tree was constructed based on the aligned amino acid sequences of 212 BAHD proteins, including 123 TwBAHDs and 89 characterized BAHDs from 31 species reported in our previous review, which have been summarized in Table S4. The results showed that these BAHD proteins were classified into six clades, and 123 TwBAHDs were distributed in five of them. Regarding TwBAHD proteins, Clade I contained sixteen members, Clade II only had one member; and there were no members TwBAHDs in Clade III. Moreover, Clade IV and Clade V had 13 and 7 TwBAHDs, respectively. Clade VI contained the largest number of TwBAHD proteins, with 86 members, 52 of which formed a specific branch that was potentially related to taxol biosynthesis. Based on phylogeny and sequence alignment, TwBAHD6, TwBAHD73, TwBAHD27, and TwBAHD83 were identified as TBT, DBAT, BAPT, and DBTNBT, respectively, the key acyltransferases on the taxol pathway, while others possessed lower sequence similarity with TAT (no more than 80%) (Figure 1).

### 2.3. Conserved Gene Structure and Protein Motif Analysis of TwBAHD Family

**Figure 2:**
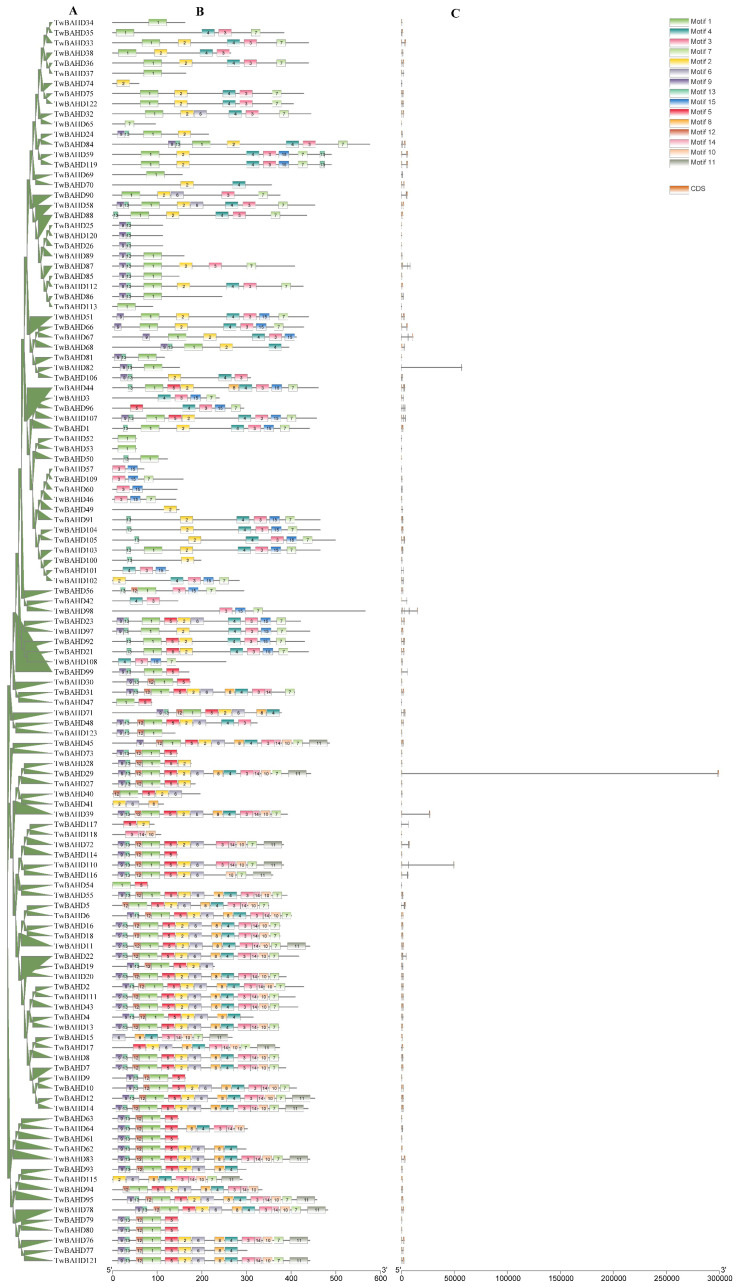
Phylogenetic tree, protein motifs, and gene structures of TwBAHDs. (**A**) Phylogenetic tree of TwBAHDs in *T. mairei*. (**B**) Fifteen conserved motifs of TwBAHD proteins, each small box indicating a motif. (**C**) The structures of introns and exons are shown in black line and yellow boxes, respectively.

As shown in Figure 2, a phylogenetic tree for 123 TwBAHD proteins was also established, followed by gene structure and protein motif analysis. The TwBAHDs were divided into five clades but were marked in a manner consistent with the phylogenetic tree in Figure 1. Within the TwBAHD genes, the number of exons ranged from 1 to 5, and genes that were clustered together generally had a high similarity in gene structure. Moreover, there was some variation among individual genes. A total of 28 intron-free *TwBAHDs* were detected, including 5 genes belonging to Clade I, 2 belonging to Clade IV, 1 belonging to Clade V, and 20 belonging to Clade VI. Most members of Clade IV had two exons and one intron, while *TwBAHD87* had five exons and four introns, which was quite different from the others.

The motif analysis of TwBAHD protein sequences was assayed using MEME, and a total of 15 motifs were predicted (Figure 2; Table S5). The results showed that most TwBAHDs contained motif 2 and (or) motif 7, corresponding to motif HXXXD and DFGWG, respectively, which was considered the signature sequence for BAHD members. Interestingly, motif 1, corresponding to motif YPLAGR, was also highly conserved in TwBAHDs. Moreover, motif 5, motif 8, motif 10, motif 11, motif 12, and motif 14 only existed in Clade VI members, suggesting that these motifs within the same subfamilies presumably performed specific functions in *Taxus*. The type and distribution of conserved motifs in the same subfamily of TwBAHDs were similar, which was consistent with the clade classification results.

### 2.4. Chromosomal Location and Duplication Events of TwBAHDs

**Figure 3:**
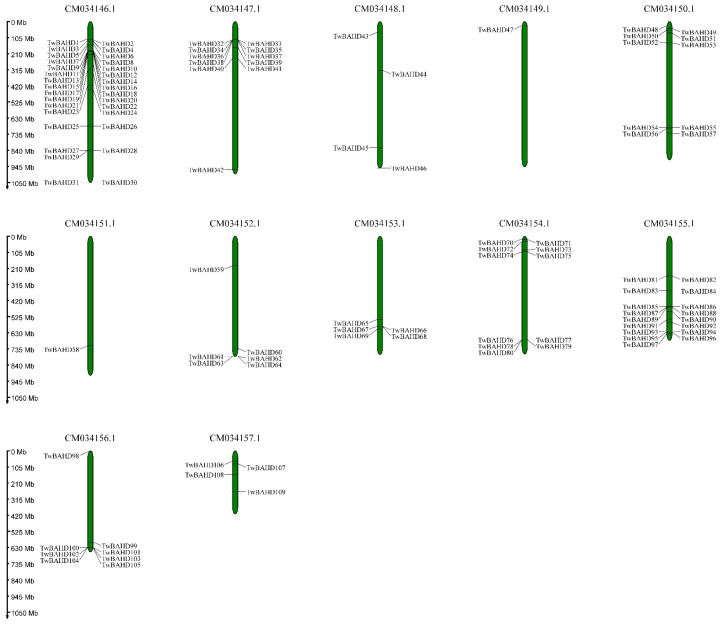
Chromosomal distribution of *TwBAHDs*.

The chromosome map of *TwBAHDs* was constructed based on the genomic sequences of *T. mairei* (Figure 3). The results showed that 109 of 123 identified *TwBAHDs* were mapped on the 12 chromosomes, designated as *TwBAHD1* to *TwBAHD109* according to the physical location, while the remaining genes (designated as *TwBAHD110* to *TwBAHD123*) were located on the contigs. Chromosome position analysis showed that these genes were unevenly distributed on all 12 chromosomes. Chromosomes 4 and 6 only contained one BAHD gene, while chromosome 1 contained the majority of BAHD members.

**Figure 4:**
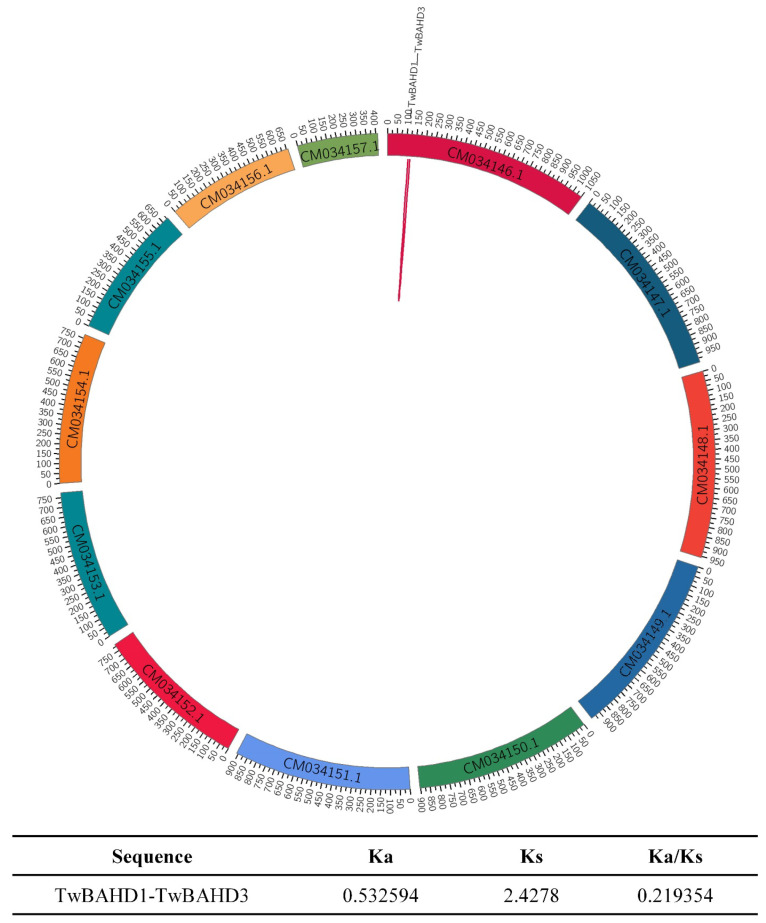
Collinearity analysis of BAHD gene family in *T. mairei* and Ka/Ks ratio values. The red line represents the homologous gene pairs in *T. mairei*.

Further research of 109 genes located on chromosomes was performed and only one possible pair of tandemly duplicated genes was found (Figure 4), namely *TwBAHD1*/*TwBAHD3*, according to the results of gene duplication analysis. Moreover, chromosome 1 was found to have a gene cluster, which was speculated to be a possible gene cluster of tandem duplication. The evolutionary selection pressure on the TwBAHD family was investigated by calculating the Ka/Ks ratio values. The results showed that the Ka/Ks ratio of the homologous gene pairs was <1.0, indicating that purifying the selection plays an important role during gene replication. The BAHD orthologous gene pairs in Taxus and Arabidopsis, as well as in Taxus and rice, were also investigated. However, no orthologs were found within these species.

### 2.5. Cis-Acting Element Analysis in the Promoters of TwBAHD Genes

**Figure 5:**
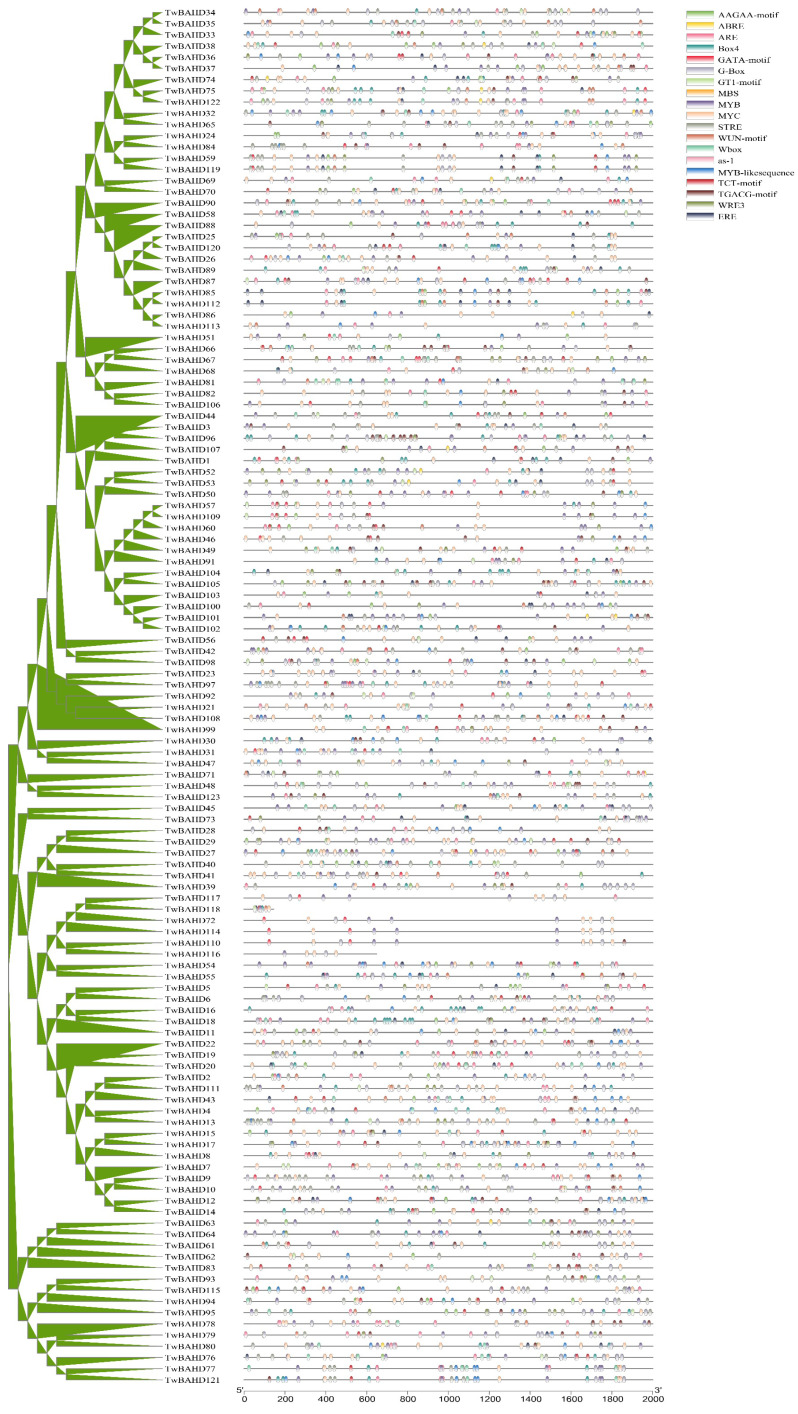
Promoter cis-regulatory element analysis of the TwBAHD gene family. Different color boxes represent different cis-regulatory elements. Some cis-regulatory element may overlap with others.

The analysis of cis-acting regulatory elements identified 20 conserved cis-acting elements in the promoter sequence of all the 123 *TwBAHDs* (Figure 5; Table S6). Among them, biotic and abiotic stress-responsive elements, including MYB, MYC, and WRE3, accounted for the largest proportion (60.8%), while light-responsiveness cis-regulatory elements were the second-largest category (21.1%), and hormone-related elements, such as ABA (ABRE), ethylene (ERE), and methyl jasmonate (MeJA) (TGACG-motif and CGTCA-motif), were also widely distributed in the promoter regions, accounting for 18.1%. Among the 20 cis-regulatory elements, ABRE and ERE were found in all five clades, while TGACG-motif and CGTCA-motif were distributed in four (absent in Clade II), which suggested a potential regulation of TwBAHD function by these hormones. In the TwBAHD family, *TwBAHD104* contained the most cis-acting elements, with a total of 67. *TwBAHD105* possessed eight TGACG motifs and CGTCA motifs.

### 2.6. Expression Profile of TwBAHD Genes by RNA-seq

**Figure 6:**
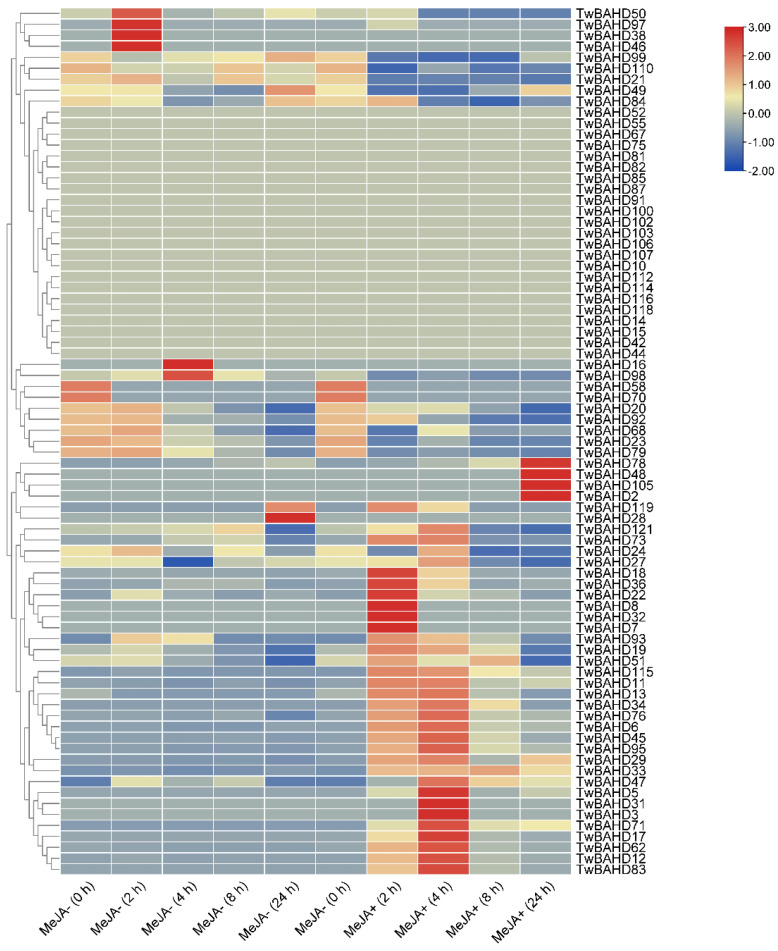
Expression profiles of BAHD acyltransferase genes in cell lines of *T. mairei* at set intervals (at 0, 2, 4, 8 and 24 h) with (+) or without (−) MeJA treatment.

The transcriptome data of the MeJA induction experiment in *T. mairei* cells were used to analyze the expression patterns of the *TwBAHD* genes. Through expression level clustering, the levels of 19 genes were significantly upregulated after 2 h of MeJA treatment; in contrast, *TwBAHD50*, *TwBAHD97*, *TwBAHD38*, and *TwBAHD46* were highly expressed in their respective no-MeJA treatments, but were significantly down-regulated after 2 h of MeJA treatment. The levels of 28 genes were significantly upregulated under treatment of MeJA+(4 h), while *TwBAHD16* and *TwBAHD98* were highly expressed in MeJA−(4 h), but were significantly down-regulated in MeJA+(4 h). *TwBAHD119* and *TwBAHD28* were highly expressed in MeJA−(24 h), but their expression levels were down-regulated in MeJA+(24 h). The levels of *TwBAHD78*, *TwBAHD48*, *TwBAHD105* and *TwBAHD2* were significantly upregulated after 24 h of MeJA treatment. Moreover, TwBAHD12, *TwBAHD24*, *TwBAHD45*, *TwBAHD95*, and *TwBAHD121* showed similar expression patterns with taxane acyltransferase genes under MeJA treatments (Figure 6).

**Figure 7:**
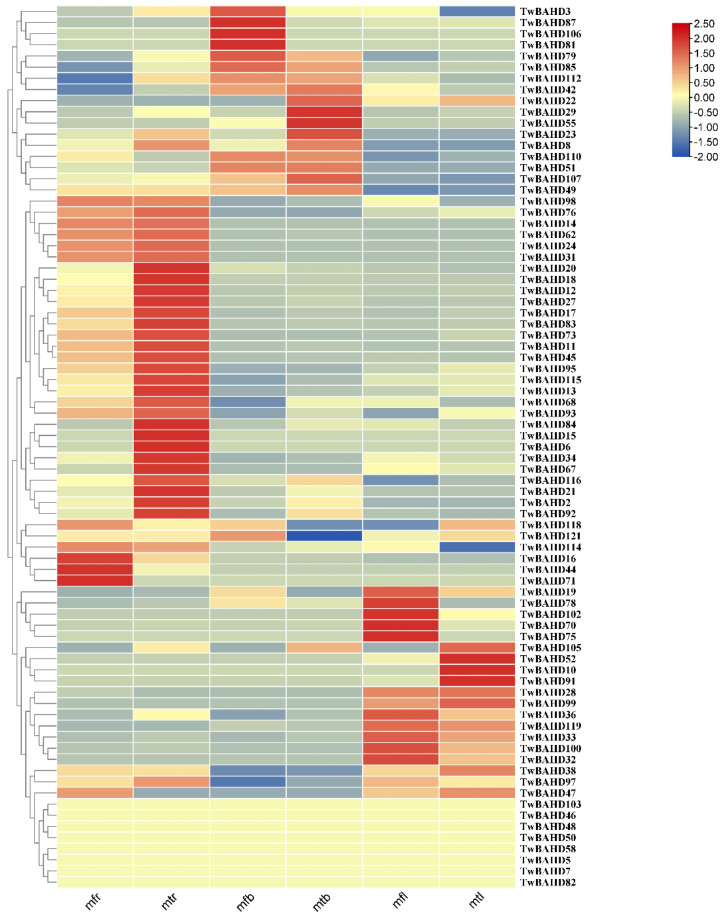
Expression profiles of BAHD acyltransferase genes in six different tissues in *T. mairei*. mfr: female root; mtr: male root; mfb: female bark; mtb: male bark; mfl: female leave; mtl: male leave.

The expression patterns of TwBAHD genes in different tissues (roots, barks, and leaves) of *T. mairei* were analyzed by previous RNA-seq data (Figure 7). The results showed that the TwBAHD genes possessed obvious tissue specificity. Among them, eight genes were not expressed in any tissues. A total of 34 genes were highly expressed in roots, while 16 were only highly expressed in males (mtr), and *TwBAHD16*, *TwBAHD44* and *TwBAHD71* were only highly expressed in females (mfr). Seventeen genes were highly expressed in barks, while *TwBAHD3*, *TwBAHD87*, *TwBAHD106*, and *TwBAHD81* were only highly expressed in mfb, and *TwBAHD29* and TwBAHD55 were only highly expressed in mtb. Nineteen genes were highly expressed in leaves, while TwBAHD19, *TwBA1D78*, *TwBAHD102*, TwBAHD70, and *TwBAHD75* were only highly expressed in mfl, and *TwBAHD105*, *TwBAHD52*, *TwBAHD10*, and *TwBAHD91* were only highly expressed in mtl. Moreover, *TwBAHD11*, *TwBAHD12*, *TwBAHD15*, and *TwBAHD17* showed similar species-specific expression patterns to those of taxane acyltransferase genes.

### 2.7. Gene Expression Analysis of TwBAHDs by qRT-PCR

**Figure 8:**
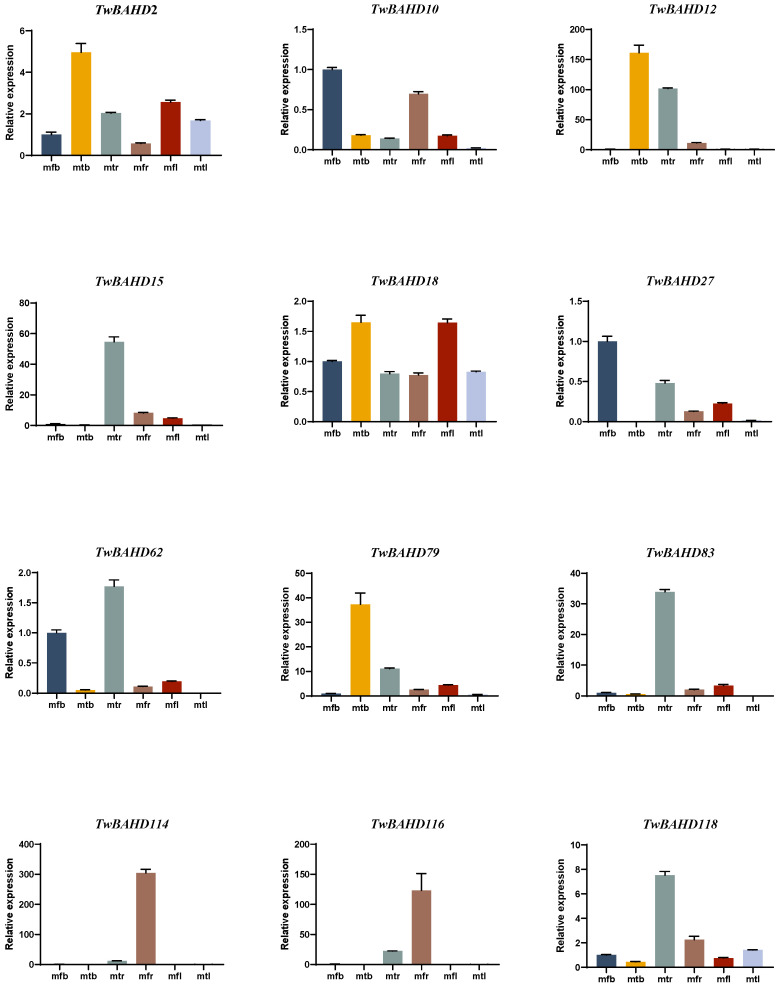
Expression of BAHD acyltransferase genes in six different tissues in *T. mairei*. Tissue-specific expression of BAHD genes in *T. mairei* was examined using qRT-PCR. The TwBAHD gene was used as an internal standard. Error bars represent standard deviations of the means of three replicates of each sample (*n* = 3). mfr: female root; mtr: male root; mfb: female bark; mtb: male bark; mfl: female leave; mtl: male leave.

The tissue-specific expression patterns of 12 TwBAHD genes from the Taxus-specific branch were randomly verified by qRT-PCR. As shown in Figure 8, the relative expression levels of *TwBAHD15*, *TwBAHD62*, *TwBAHD83* and *TwBAHD118* in mtr and *TwBAHD114* and TwBAHD116 in mfr were significantly higher than those in other tissues. *TwBAHD2*, *TwBAHD12*, *TwBAHD18* and *TwBAHD79* showed higher relative expression levels in mtb, while *TwBAHD10* and *TwBAHD27* showed higher levels in mfb. Notably, *TwBAHD18* was highly expressed in both mtb and mfl.

### 2.8. Content of Taxanes in Different Tissues of T. mairei

**Figure 9:**
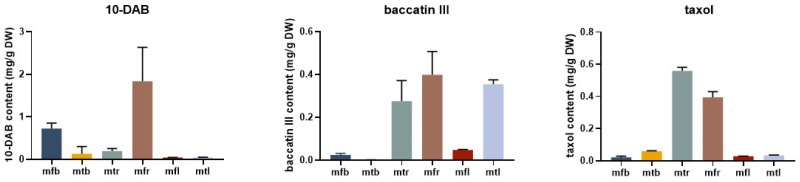
Content of 10-DAB, baccatin III, and taxol in different tissues of *T. mairei*. Error bars represent standard deviations of the means of three replicates of each sample (*n* = 3). mfr: female root; mtr: male root; mfb: female bark; mtb: male bark; mfl: female leave; mtl: male leave.

As shown in Figure 9, the content of different taxanes showed obvious tissue specificity in *T. mairei*. Among them, the content of 10-DAB ranged from 0.0314 to 1.8398 mg/g, while its highest level was observed in mtr. The level of baccatin III accumulated in different tissues ranged from 0.0010 to 0.3993 mg/g, with the highest level being observed in mfr. For taxol, the highest content was detected in mtr, up to 0.5585 mg/g, while the lowest content was in mfb, about 0.0218 mg/g. Overall, 10-DAB, baccatin III and taxol were mainly accumulated in the roots of *T. mairei*; the former two also have higher content in male leaves and female barks, respectively.

### 2.9. Correlation Analysis between TwBAHD Gene Expression and Taxane Levels

**Figure 10:**
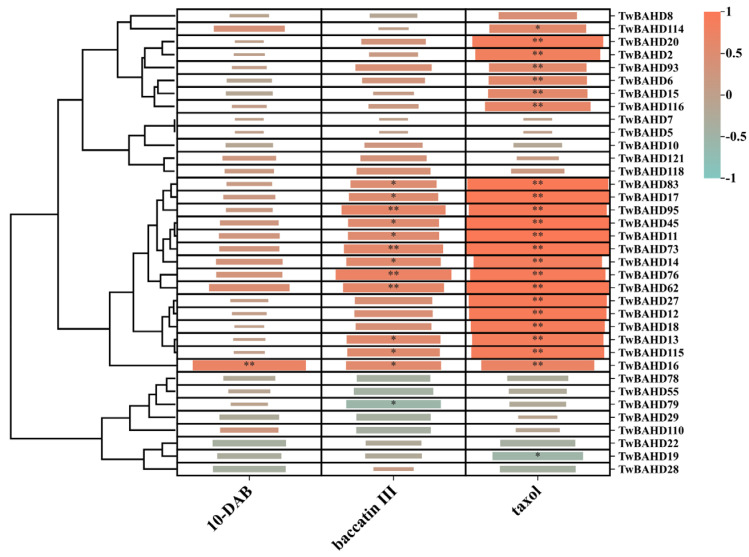
Correlation analysis between TwBAHD gene expression and taxane levels. Shown are the heatmap of correlation coefficient between Taxus-specific TwBAHD genes and representative taxanes in *T.mairei*. * indicates significant correlations at *p* < 0.05 level; ** indicates significant correlations at *p* < 0.01 level.

The correlation analysis was performed to evaluate the relationships between Taxus-specific TwBAHD gene expression and taxane levels within different tissues of *T. mairei* (Figure 10). In this study, 10-DAB was highly positively correlated with *TwBAHD16* (*p* < 0.01); baccatin III was highly positively correlated with *TwBAHD95*, *TwBAHD73*, *TwBAHD76*, and *TwBAHD62* (*p* < 0.01); and taxol was highly positively correlated with 21 TwBAHD genes (*p* < 0.01). Moreover, *T. mairei* demonstrated positive correlations between baccatin III and 8 TwBAHD genes (*p* < 0.05), as well as taxol and *TwBAHD114* (*p* < 0.05), and negative correlations between baccatin III and *TwBAHD79* (*p* < 0.05), as well as taxol and *TwBAHD19* (*p* < 0.05).

### 2.10. 3D Modeling of TwBAHD76 and TwBAHD121

**Figure 11:**
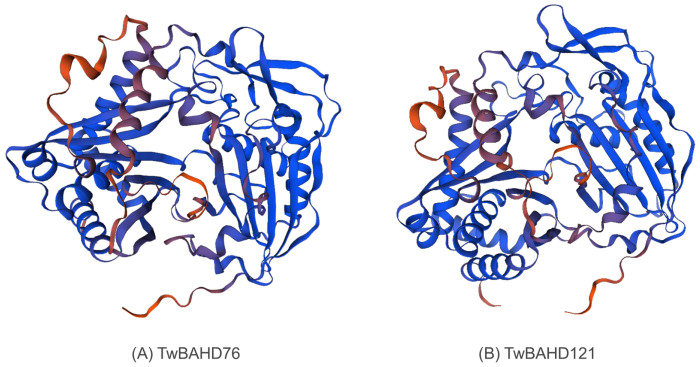
The 3D models of selected TwBAHDs constructed using the SWISS-MODEL. (**A**) The 3D model of TwBAHD76. (**B**) The 3D model of TwBAHD121.

To understand the protein differences among TAT and selected TwBAHDs, we used SWISS-MODEL to model TwBAHD76 and TwBAHD121 (Figure 11). The results showed that both TwBAHD76 and TwBAHD121 are transferases; the homology of the tertiary structure of TwBAHD76 and TwBAHD121 constructed by the A0A0M4G1D0.1.A (Taxadien-5-alpha-ol-O-acetyltransferase) model was 89.14% and 99.32%, respectively, with more α-helices and random coils. These results further suggest that TwBAHD76 and TwBAHD121 function as TAT activity.

### 2.11. Subcellular Localization of TwBAHD76 and TwBAHD121 Genes

**Figure 12:**
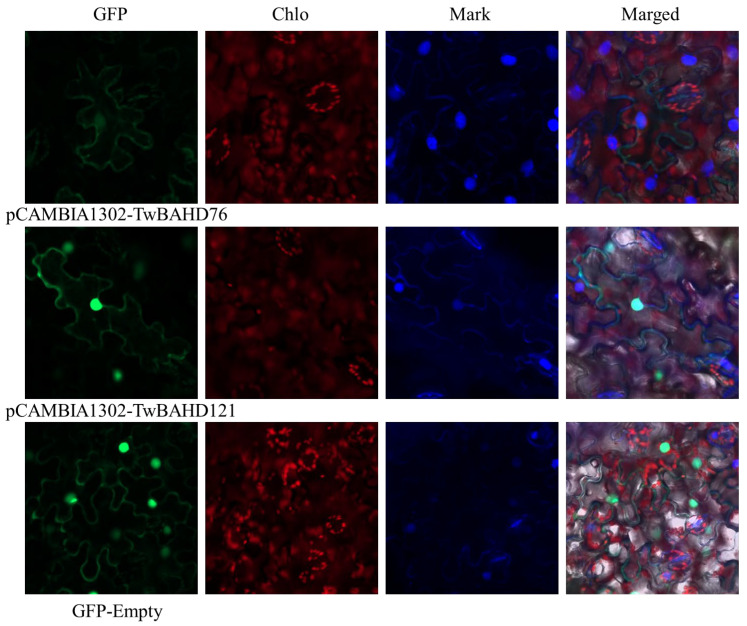
Subcellular localization of TwBAHD76 and TwBAHD121 proteins. Selected TwBAHD-GFP fusion protein and GFP-Empty as the control were independently transiently expressed in tobacco leaves and imaged under a confocal microscope. Bars = 10 μm.

The *TwBAHD76* and *TwBAHD121* genes were fused into the N-terminal of GFP to construct vector pCAMBIA1302-*TwBAHD76* and pCAMBIA1302-*TwBAHD121*, respectively. The transient expression of tobacco leaves was analyzed with the *A. tumefaciens* method, and the fluorescence signal was observed by a laser confocal microscope. It was observed that the green fluorescence signal mainly existed in the cell nucleus and cell periphery in both constructed vectors, while empty 1302-GFP was observed in the nucleus, intercellular and cell membrane (Figure 12), indicating that both *TwBAHD76* and *TwBAHD121* underwent functional and morphological changes in the nucleus and cell membrane. These observations established the potential role of TwBAHD76 and TwBAHD121 proteins in the taxol pathway and their influence on taxane intermediate synthesis.

## 3. Discussions

The BAHD acyltransferases possess a huge and diverse family in plants, and numerous studies have reported that BAHD members are involved in multiple biological processes in plant growth, development, and stress responses [31], and they also showed distinct patterns of evolution in particular species, such as the *Taxus* [32]. However, a systematic characterization of BAHDs in the *Taxus* species has not been performed. In this study, the genome-wide identification and characterization of BAHD family members in *T. mairei* were carried out. A total of 123 TwBAHDs have been identified and divided into five clades based on phylogeny and conserved motif analyses.

There were no more than five BAHDs in the common ancestor of land plants and algae. The BAHD family expanded significantly during plant evolution, which also resulted in plant functional and metabolic diversity [33]. Genome-wide identification of the BAHD family has been reported in many model plants such as Arabidopsis [34], barley [25], rice [35], and poplar [36]. In comparison, *Taxus mairei* possessed more BAHD acyltransferase genes than Arabidopsis, but had similar numbers to those of barley, rice, and poplar. Based on the phylogeny, most of the TwBAHD members in Clade I are the likely orthologs of enhanced pseudomonas susceptibility1 (EPS1), which is involved in salicylic acid biosynthesis [37], while TwBAHD24, TwBAHD59, TwBAHD84, and TwBAHD119 potentially have malonyltransferase or aromatic acyltransferase activity, participating in the acylation of anthocyanins and phenolic glucosides. In Clade V, the TwBAHDs function as hydroxycinnamoyl transferase, which is involved in lignin synthesis, mediating plant growth and development [38]. In Clade VI, TwBAHD21, TwBAHD92, and TwBAHD108 are orthologs of aliphatic suberin feruloyl transferase, feruloyltransferase, and deficient in cutin ferulate [39]; also in this clade, TwBAHD23, TwBAHD42, TwBAHD56, TwBAHD97, and TwBAHD98 are orthologs of alcohol acyltransferase, which is required to produce volatile esters [40]. However, in most cases, the clade members of *T. mairei* clearly possess their own branch in phylogeny compared with the identified members from other species, which further indicated that these TwBAHDs may also be functionally different from orthologs in other species. Moreover, the number of TwBAHDs in different clades was also different from that of other species. For example, Clade II only contained one member in *T. mairei*, which could be the orthologous gene of ECERIFERUM2 [41]. Clade III did not include the BAHD gene of *T. mairei*. Clade VI, containing 86 TwBAHD genes, was the largest clade, which also contained a Taxus-specific branch with 52 genes related to taxol biosynthesis. These combined results indicated that the BAHD family plays an important role in plant evolution, while the BAHD genes could have been either acquired and expanded or specially lost in *T. mairei* during the evolution process. Notably, the number of TwBAHD genes reported in this study was inconsistent with the preliminary results by Xiong et al. [30], who reported 127 TwBAHDs with 53 members related to taxol biosynthesis. Considering the loss of several TwBAHDs, including TAT, a key enzyme in the taxol pathway [42], further optimization and improvement are very necessary for the genomic data of *T. mairei* in the future.

Gene duplications, including whole-genome duplication (WGD), tandem duplication (TD), chromosomal segmental duplication (SD), and retrotransposition (TRD), are considered the main driving forces in the evolution of genomes and genetic systems [43,44,45]. Previous studies have reported that no recent WGD event occurred in the *T. mairei* genome, while *Taxus* only shared the common ancient WGD with other coniferophyte lineages [30]. In this study, the duplicate genes in the *T. mairei* genome were analyzed, and only one pair of duplicate genes (*TwBAHD1*/*TwBAHD3*) were found on chromosome 1 with Ka/Ks ratios < 1. This indicated that the TD contributed to the expansion of TwBAHD family genes [46], and the duplicate genes have undergone purifying selection without serious functional differentiation [47]. Moreover, multiple TwBAHD members of a subfamily were also observed to cluster in the same or adjacent intergenic regions on a single chromosome, which were probably potential tandem duplicate genes. The results in this study were not consistent with those reported by Tuominen et al. [48], who observed a large number of SD events in the *Hordeum vulgare* BAHD family. This indicated a species-specific expansion of BAHD genes. A previous study has proven that secondary metabolic genes are more likely than the average to experience lineage-specific diversification via TD events [47]. Due to evolutionary constraints for balanced stoichiometry, enzymes acting at different locations in the same pathway would likely retain a specific number of gene copies across the taxa [49,50,51,52]. This could explain the species-specific expansion patterns occurred in *T. mairei*.

Gene structure variation plays an important part in gene evolution. The exon/intron composition analysis showed that the number of exons varied from 1 to 5 for all TwBAHD genes, while most of the *TwBAHDs* only possess one or two introns. Moreover, the TwBAHD members within the same subfamily shared similar exon/intron patterns. These observations were consistent with previous findings in other plants [53]. The conserved motifs further reveal the grouping specificity of motif distribution of *T. mairei*. The two known conserved motifs, the HXXXD and DFGWG, are involved in the binding of acyl-CoA donor and the structural integrity of the enzyme–donor complex in the BAHD family [54,55,56]. They are also highly conserved in the TwBAHD family, which has been identified to contain at least one of the two conserved motifs in the amino acid sequences of family members. Interestingly, the YPLAGR motif was also conserved in multiple clades of *T. mairei*. This motif was first reported in the *Populus paralogues* BAHD (PpBAHD) family, which corresponds to a small α-helix-3 on vinorine synthase, but there has been no mutagenesis analysis for this region [57]. Its lower conservation in Clade Ia of the PpBAHD family was associated with a lack of this α-helix and an extra string of 9–14 residues corresponding to Gly and Arg residues of this motif on vinorine synthase [58]. For TwBAHDs, this motif was also less conserved in Clade II and individual subfamily members of Clade VI. Moreover, the clade-specific motifs in the TwBAHD family should be of value in future studies to understand the diverse enzyme functions.

The cis-regulatory element analysis can potentially reveal the gene function of *TwBAHDs*. In this study, the upstream promoter of BAHD genes in *T. mairei* mainly contained three types of cis-elements, including stress-, light-, and hormone-responsive elements, which indicated that the TwBAHD genes are likely to participate in responses against various adverse environmental stresses. This was consistent with findings in other species [59]. Cis-regulatory elements related to ABA and ethylene are frequently observed in promoter sites, indicating that TwBAHD genes are more induced by hormones associated with the response to stress stimuli, and thus play a role in the regulation of plant structure and function, cell stability, and the production of secondary metabolites [16]. The response of plants to biotic and abiotic stresses involves a very complex network of gene regulation, and this process is usually mediated by multiple transcription factors. The presence of transcription factor binding sites related to MYB and MYC in TwBAHDs further suggested that transcription factor-based signal transduction networks were crucial in regulating the response of *T. mairei* to stress [60]. Moreover, previous studies also reported the critical role of BAHD acyltransferases in response to fungal infection [61]. These combined results showed that promoters could control structural and morphological changes in plant–environment interactions to adapt to unfavorable external environments. Notably, we identified several TGACG and CGTCA motifs, which mainly function as MeJA response elements. Previous studies have reported that MeJA treatment could increase the accumulation of alkaloids and phenolic substances in plants and, in the case of *Taxus*, is also key to regulating taxol levels [62,63]. In the present study, RNA-seq data of MeJA treatment in *T. mairei* cells showed that most of the TwBAHD genes respond positively to MeJA within 24 h of treatment, which was basically consistent with BAHD function in the secondary metabolism. The expression levels of identified key acyltransferase genes on the taxol pathway were up-regulated 2–4 h after MeJA treatment, which provided a basis for MeJA-mediated increase in taxol content in *Taxus*. Simultaneously, the TwBAHD members, which showed similar expression patterns and were clustered in the same subfamily with those taxane acyltransferase genes, are considered newly candidate genes for taxol biosynthesis. Certainly, further experiments are needed to ascertain which members are associated with taxol biosynthesis and whether this directly coincides with the presence of cis-elements.

In this study, the accumulation of taxanes in *T. mairei* showed obvious tissue specificity, while a similar pattern was also observed in the expressions of TwBAHD genes. The qRT-PCR results further confirmed the expression difference of representative TwBAHD genes in the species-specific branch. According to the results of correlation analysis, the levels of many *TwBAHDs* in the Taxus-specific branch were significantly associated with the accumulation of taxanes, which provided important clues for further screening candidate acyltransferase genes involved in the taxol pathway. The high levels of taxane acyltransferase genes in the roots of male plants also provided a reasonable explanation for the high content of taxol in the roots of *T. mairei* [64]. Interestingly, the TwBAHD genes that exhibited consistent expression patterns with taxane acyltransferase genes in MeJA induction and tissue-specific expression experiments were different and overlapped only in a small part. This indicated diverse and complex accumulation patterns of taxol in *Taxus* in response to environmental changes. Moreover, the TAT showed relatively low sequence similarity compared with TwBAHDs in Taxus-specific branches; however, the tertiary structures of TwBAHD121 and TwBAHD76 were found to be 99% and 89% similar with that of TAT, suggesting that they may function as TAT enzymes. This was consistent with the results reported by Xiong et al. [30]. TAT has no organelle-targeting sequences, thus suggesting a cytosolic localization [65], while subcellular localization results showed that both *TwBAHD121* and *TwBAHD76* genes were localized in the cell nucleus and cell periphery. We do not rule out the possibility that these two TwBAHD-mediated acylation steps help to control flux through the pathway to produce taxoid variants likely also possessing distinct biological roles [66]. Subsequent studies should focus on the in vivo and in vitro functional validation of these TwBAHDs, which will provide new insights into taxol biosynthesis.

## 4. Materials and Methods

### 4.1. Plant Materials

Trees of *T. mairei* were grown in the arboretum of Nanjing botanical garden Mem. Sun Yat-Sen (32°3′ N, 118°49′ E). For samples used for quantitative real-time PCR, young leaves, roots, and barks were collected from male and female trees with the same tree ages and relatively consistent growth conditions. Three biological replicates were prepared, whereas each of samples was from three individual plants. All samples were immediately frozen in liquid nitrogen and stored at −80 °C until used.

### 4.2. Identification of BAHD Family Members in Taxus mairei

In this study, the genome of *T. mairei* was obtained from the NCBI (https://www.ncbi.nlm.nih.gov/datasets/genome/GCA_019776745.2/, accessed on 31 November 2022). To identify members of the BAHD family in *T. mairei*, the Hidden Markov Model (HMM) file of the BAHD acyltransferase domain (PF02458) was downloaded from the protein family (Pfam) (https://www.ncbi.nlm.nih.gov/datasets/genome/GCA_019776745.2/, accessed on 31 November 2022) database, and used to query the *T. mairei* genome database with an E-value threshold of 1 × 10^−5^ of HMMER 3.0 to search for candidate BAHDs [67]. These candidate BAHDs were further verified by the SMART database (http://smart.embl-heidelberg.de/, accessed on 30 June 2023) and NCBI conservative domain database (https://www.ncbi.nlm.nih.gov/cdd, accessed on 30 June 2023) [68,69]. Then, all the identified BAHDs were named by adding a prefix of “Tw” for *Taxus wallichiana* and followed by Arabic numbers serially starting from 1. Their physical and chemical characteristics, including amino acid number, molecular weight (MW), isoelectric point (pI), hydrophobicity, and instability index were analyzed using Prosite ExPASy server (http://web.expasy.org/protparam/, accessed on 31 May 2023), and subcellular localizations were predicted by the online software WoLF PSORT (https://wolfpsort.hgc.jp/, accessed on 30 June 2023) [70]. Moreover, three-dimensional (3D) modeling of representative TwBAHD proteins was performed using the SWISS-MODEL with default settings (https://swissmodel.expasy.org/, accessed on 30 June 2023).

### 4.3. Phylogenetic Analysis

Multiple amino acid sequence alignment of TwBAHDs was performed using mafft (v7.427) with default parameters, and the output data were saved in MEGA format. The phylogenetic tree was constructed with the identified TwBAHDs and representative BAHDs from our previous reported review [23] by MEGA10 software (version 10.0.2) using the maximum likelihood (ML) method with the optimal replacement model and 1000 bootstrap replications [71]. The constructed phylogenetic tree was optimized by iTOL v6 (https://itol.embl.de/, accessed on 30 June 2023) [72].

### 4.4. Analysis of Gene Structures and Conserved Motifs

The positions of exons and introns of each TwBAHD gene were obtained from genome of *T. mairei*. Conserved motifs in the TwBAHD proteins were analyzed by MEME suite (http://meme-suite.org/tools/meme, accessed on 30 June 2023) [73] with the following parameters: maximum motif number was 15; minimum motif width was 6; maximum motif width was 100; and the distribution of motif occurrences was zero or one per sequence. The motif corresponding to the pfam domain was indicated by the Pfam database. The gene structures and protein motifs were visualized by TBtools software (version 1.108) [74].

### 4.5. Chromosomal Distribution and Synteny Analysis

The chromosome location of each identified TwBAHD gene was retrieved from the genome of *T. mairei*. Then, the chromosome location map was visualized using the MG2C server (http://mg2c.iask.in/mg2c_v2.1/, accessed on 31 May 2023) [75]. Homologous gene pairs and syntenic relationships of BAHD family genes in *T. mairei* were identified using Multiple Collinearity Scan toolkit (MCScanX) (https://github.com/wyp1125/MCScanX, accessed on 25 March 2024) with default parameters. The Ka/Ks calculator 2.0 was used to calculate the ratio between the non-synonymous rate (Ka) and the synonymous substitution rate (Ks) of homologous genes [76].

### 4.6. Analysis of Promoter Cis-Regulatory Element

A 2000 bp sequence upstream of the translation start site of TwBAHD genes was extracted from the genome of *T. mairei* as the promoter sequence, and its cis-regulatory elements were predicted using PlantCARE (https://bioinformatics.psb.ugent.be/webtools/plantcare/html/, accessed on 31 May 2023) [77]. The results were visualized using TBtools software (version 1.108).

### 4.7. Expression Patterns of the TwBAHD Gene Family Based on RNA-Seq Databases

The transcriptome databases of *T.mairei* cell lines under MeJA treatment (0 h, 2 h, 4 h, 8 h, and 24 h) [30] were used to analyze the induction expression patterns of the TwBAHD family. Moreover, the RNA-Seq data of six different tissues of *T.mairei*, including female root (mfr), male root (mtr), female bark (mfb), male bark (mtb), female leaf (mfl), and male leaf (mtl) [30], were used to analyze the tissue expression patterns of the TwBAHD family. All the data were mapped to the *T.mairei* genome with TwBAHD genes as the queries. According to the BLAST search, genes from transcriptome that were more than 90% similar with identified TwBAHD members were selected, and gene expression levels were represented by fragments per kilobase of exon per million fragments mapped (FPKM). Heat maps were plotted using TBtools software (version 1.108).

### 4.8. RNA Extraction and qRT-PCR Analysis

Total RNA was extracted from various tissues of *T.mairei* using Plant RNA Extraction Kit (Huayueyang, Beijing, China) following the manufacturer’s protocol. The first-strand cDNA was synthesized by PrimeScript RT reagent Kit with gDNA Eraser (TaKaRa, Dalian, China). Then, real-time quantitative PCR (qRT-PCR) was performed by SYBR Premix Ex TaqTM Kit (TaKaRa, Dalian, China) in 20 μL reaction volumes using ABI StepOne Plus system (Thermo, MA, USA). The program was conducted as follows: 90 °C for 30 s, followed by 40 cycles of 95 °C for 5 s and 60 °C for 30 s in 96-well optical reaction plates. Three replicates were performed for each selected gene. The actin was taken as a reference gene. The relative expression levels were calculated by the 2^−ΔΔCt^ method [78]. Primers used in this study were designed by Primer5.0 [79], and are listed in Table S1.

### 4.9. Taxanes’ Determination and Correlation Analysis

The powder (0.2 g) from mfr, mtr, mfb, mtb, mfl, and mtl of *T. mairei* was weighed with high precision and added to 3 mL methanol for ultrasonic extraction using an ultrasonic apparatus (KQ5200DE, Kunshan Ultrasound Instrument Company, Kunshan, China) according to the method of Li et al. [80]. The sample was then centrifuged at 10,000 rpm for 7 min to collect the supernatant. After drying using a nitrogen-blowing instrument, the dried residue was dissolved in 1 mL of methanol solution and was filtered by a 0.22 μm microfiltration membrane. The taxanes’ determination was carried out using Agilent 1100 high-performance liquid chromatography (HPLC) and an Agilent diode array detector (DAD). The separation of taxanes was achieved on a penomenex Curosil-PFP column (250 mm × 4.6 mm, 5 μm). The mobile phase was composed of methanol and water through the following gradient eluted program: 0–2 min, 35:65 (*v*/*v*); 2–6 min, 55:45 (*v*/*v*); 6–24 min, 65:35 (*v*/*v*); 24–40 min, 100:0 (*v*/*v*); 40–46 min, 35:65 (*v*/*v*). The detection wavelength was 227 nm with the flow rate of 1 mL/min under 40 °C, and an injection volume of 10 μL. A mixed standard solution of taxanes was employed to create a standard curve for quantification. The standards, including 10-DAB (≥98%, CAS: 32981–86–5), baccatin III (≥98%, CAS: 27548–893–2), and taxol (≥99.9%, CAS: 33069–62–4), were purchased from the National institutes for food and drug control (Beijing, China). Moreover, correlation analysis was performed to reveal a correlated degree between the expression of Taxus-specific TwBAHD genes and the levels of taxanes in different tissues of *T. mairei* by using an online tool ChiPlot (https://www.chiplot.online/, accessed on 31 May 2023).

### 4.10. Subcellular Localization of the TwBAHD Genes

The fusion constructs of BAHD and GFP were set up to confirm the subcellular localization of TwBAHD76 and TwBAHD121. Primer sequences are provided in Table S1. The amplified fragment was introduced into the pCAMBIA1302 to generate the recombinant expression vector pCAMBIA1302-TwBAHD76 and pCAMBIA1302-TwBAHD121. Then, the vectors were transformed into *Agrobacterium tumefaciens* strain GV3101, and infiltrated into leaves of 4-week-old tobacco (*Nicotiana benthamiana*) plants using an *A. tumefaciens* method. After dark conditions for two days, the injected leaves were mounted in water and observed through a laser confocal microscopy (Zeiss LSM900, Jena, Germany) with empty 1302-GFP as a control.

## 5. Conclusions

In this study, 123 TwBAHD genes were identified in the *T. mairei* genome and were classified into six clades based on sequence similarity and phylogenetic relationships. Conserved motifs, gene structure, and evolutionary relationships of TwBAHD genes were established and analyzed, while a Taxus-specific branch containing 52 genes was related to taxol biosynthesis. The investigation of the cis-regulatory elements of TwBAHD genes indicated that many TwBAHD genes involved responses to biotic and abiotic stresses, which was consistent with BAHD function in the secondary metabolism. RNA-seq analysis comprehensively revealed multiple expression patterns of TwBAHD genes, and a series of candidate genes potentially associated with taxol biosynthesis could provide a basis for the subsequent functional characterization and future improvement of mass production of taxol by genetic engineering.

## Data Availability

Data is contained within the article and Supplementary Material.

## Supplementary Materials

The following supporting information can be downloaded at: https://www.mdpi.com/article/10.3390/ijms25073777/s1.

## References

[1] Riffi O., reda Kachmar M., M’Hamdi Z., Fliou J., Chakir S., Amechrouq A. (2023). Study of the chemical composition and evaluation of the antioxidant and antimicrobial activity of *Taxus baccata* L.. Arab. J. Chem..

[2] Alqahtani F.Y., Aleanizy F.S., El Tahir E., Alkahtani H.M., AlQuadeib B.T. (2019). Paclitaxel. Profiles DrugSubst. Excip. Relat. Methodol..

[3] Croteau R., Ketchum R.E., Long R.M., Kaspera R., Wildung M.R. (2006). Taxol biosynthesis and molecular genetics. Phytochem. Rev. Proc. Phytochem. Soc. Eur..

[4] Bocci G., Di Paolo A., Danesi R. (2013). The pharmacological bases of the antiangiogenic activity of paclitaxel. Angiogenesis.

[5] Sabzehzari M., Zeinali M., Naghavi M.R. (2020). Alternative sources and metabolic engineering of Taxol: Advances and future perspectives. Biotechnol. Adv..

[6] Wang T., Li L., Zhuang W., Zhang F., Shu X., Wang N., Wang Z. (2021). Recent Research Progress in Taxol Biosynthetic Pathway and Acylation Reactions Mediated by Taxus Acyltransferases. Molecules.

[7] Walker K., Croteau R. (2000). Taxol biosynthesis: Molecular cloning of a benzoyl-CoA:taxane 2alpha-O-benzoyltransferase cDNA from taxus and functional expression in *Escherichia coli*. Proc. Natl. Acad. Sci. USA.

[8] Walker K., Fujisaki S., Long R., Croteau R. (2002). Molecular cloning and heterologous expression of the C-13 phenylpropanoid side chain-CoA acyltransferase that functions in Taxol biosynthesis. Proc. Natl. Acad. Sci. USA.

[9] Walker K., Long R., Croteau R. (2002). The final acylation step in taxol biosynthesis: Cloning of the taxoid C13-side-chain N-benzoyltransferase from Taxus. Proc. Natl. Acad. Sci. USA.

[10] Lin S.L., Wei T., Lin J.F., Guo L.Q., Wu G.P., Wei J.B., Huang J.J., Ouyang P.L. (2018). Bio-production of Baccatin III, an Important Precursor of Paclitaxel by a Cost-Effective Approach. Mol. Biotechnol..

[11] Jiménez-Barbero J., Amat-Guerri F., Snyder J.P. (2002). The solid state, solution and tubulin-bound conformations of agents that promote microtubule stabilization. Curr. Med. Chem. Anti-Cancer Agents.

[12] Jones P., Vogt T. (2001). Glycosyltransferases in secondary plant metabolism: Tranquilizers and stimulant controllers. Planta.

[13] Sah B., Subban K., Jayabaskaran C. (2019). Biochemical insights into the recombinant 10-deacetylbaccatin III-10-β-O-acetyltransferase enzyme from the Taxol-producing endophytic fungus Lasiodiplodia theobromae. FEMS Microbiol. Lett..

[14] D’Auria J.C. (2006). Acyltransferases in plants: A good time to be BAHD. Curr. Opin. Plant Biol..

[15] Sharma S., Khare P., Kumar A., Chunduri V., Kumar A., Kapoor P., Mangal P., Kondepudi K.K., Bishnoi M., Garg M. (2020). Anthocyanin-Biofortified Colored Wheat Prevents High Fat Diet-Induced Alterations in Mice: Nutrigenomics Studies. Mol. Nutr. Food Res..

[16] St-Pierre B., De Luca V., Romeo J.T., Ibrahim R., Varin L., De Luca V. (2000). Chapter Nine—Evolution of Acyltransferase Genes: Origin and Diversification of the BAHD Superfamily of Acyltransferases Involved in Secondary Metabolism. Recent Advances in Phytochemistry.

[17] Dudareva N., D’Auria J.C., Nam K.H., Raguso R.A., Pichersky E. (1998). Acetyl-CoA:benzylalcohol acetyltransferase—An enzyme involved in floral scent production in Clarkia breweri. Plant J. Cell Mol. Biol..

[18] St-Pierre B., Laflamme P., Alarco A.M., De Luca V. (1998). The terminal O-acetyltransferase involved in vindoline biosynthesis defines a new class of proteins responsible for coenzyme A-dependent acyl transfer. Plant J. Cell Mol. Biol..

[19] Fujiwara H., Tanaka Y., Yonekura-Sakakibara K., Fukuchi-Mizutani M., Nakao M., Fukui Y., Yamaguchi M., Ashikari T., Kusumi T. (1998). cDNA cloning, gene expression and subcellular localization of anthocyanin 5-aromatic acyltransferase from Gentiana triflora. Plant J. Cell Mol. Biol..

[20] Wang L., Chen K., Zhang M., Ye M., Qiao X. (2022). Catalytic function, mechanism, and application of plant acyltransferases. Crit. Rev. Biotechnol..

[21] Fayad M.A., Charles S., Shelvy S., Sheeja T.E., Sangeetha K., Angadi U.B., Tandon G., Iquebal M.A., Jaiswal S., Kumar D. (2024). Whole genome based identification of BAHD acyltransferase gene involved in piperine biosynthetic pathway in black pepper. J. Biomol. Struct. Dyn..

[22] Yu X.H., Gou J.Y., Liu C.J. (2009). BAHD superfamily of acyl-CoA dependent acyltransferases in Populus and Arabidopsis: Bioinformatics and gene expression. Plant Mol. Biol..

[23] Xu D., Wang Z., Zhuang W., Wang T., Xie Y. (2023). Family characteristics, phylogenetic reconstruction, and potential applications of the plant BAHD acyltransferase family. Front. Plant Sci..

[24] Xia Y., Nikolau B.J., Schnable P.S. (1997). Developmental and hormonal regulation of the arabidopsis CER2 gene that codes for a nuclear-localized protein required for the normal accumulation of cuticular waxes. Plant Physiol..

[25] Yamane M., Takenoya M., Yajima S., Sue M. (2020). Crystal structure of barley agmatine coumaroyltransferase, an N-acyltransferase from the BAHD superfamily. Acta Crystallogr. Sect. F Struct. Biol. Commun..

[26] Yamane M., Takenoya M., Yajima S., Sue M. (2021). Molecular and structural characterization of agmatine coumaroyltransferase in Triticeae, the key regulator of hydroxycinnamic acid amide accumulation. Phytochemistry.

[27] Kage U., Karre S., Kushalappa A.C., McCartney C. (2017). Identification and characterization of a fusarium head blight resistance gene TaACT in wheat QTL-2DL. Plant Biotechnol. J..

[28] Burhenne K., Kristensen B.K., Rasmussen S.K. (2003). A new class of N-hydroxycinnamoyltransferases. Purification, cloning, and expression of a barley agmatine coumaroyltransferase (EC 2.3.1.64). J. Biol. Chem..

[29] Moglia A., Acquadro A., Eljounaidi K., Milani A.M., Cagliero C., Rubiolo P., Genre A., Cankar K., Beekwilder J., Comino C. (2016). Genome-Wide Identification of BAHD Acyltransferases and In vivo Characterization of HQT-like Enzymes Involved in Caffeoylquinic Acid Synthesis in Globe Artichoke. Front. Plant Sci..

[30] Xiong X., Gou J., Liao Q., Li Y., Zhou Q., Bi G., Li C., Du R., Wang X., Sun T. (2021). The Taxus genome provides insights into paclitaxel biosynthesis. Nat. Plants.

[31] Bartley L.E., Peck M.L., Kim S.R., Ebert B., Manisseri C., Chiniquy D.M., Sykes R., Gao L., Rautengarten C., Vega-Sánchez M.E. (2013). Overexpression of a BAHD acyltransferase, OsAt10, alters rice cell wall hydroxycinnamic acid content and saccharification. Plant Physiol..

[32] Kusano H., Li H., Minami H., Kato Y., Tabata H., Yazaki K. (2019). Evolutionary Developments in Plant Specialized Metabolism, Exemplified by Two Transferase Families. Front. Plant Sci..

[33] Kuang X., Sun S., Wei J., Li Y., Sun C. (2019). Iso-Seq analysis of the *Taxus cuspidata* transcriptome reveals the complexity of Taxol biosynthesis. BMC Plant Biol..

[34] Moghe G., Kruse L.H., Petersen M., Scossa F., Fernie A.R., Gaquerel E., D’Auria J.C. (2023). BAHD Company: The Ever-Expanding Roles of the BAHD Acyltransferase Gene Family in Plants. Annu. Rev. Plant Biol..

[35] Peng M., Gao Y., Chen W., Wang W., Shen S., Shi J., Wang C., Zhang Y., Zou L., Wang S. (2016). Evolutionarily Distinct BAHD N-Acyltransferases Are Responsible for Natural Variation of Aromatic Amine Conjugates in Rice. Plant Cell.

[36] de Vries L., MacKay H.A., Smith R.A., Mottiar Y., Karlen S.D., Unda F., Muirragui E., Bingman C., Vander Meulen K., Beebe E.T. (2022). pHBMT1, a BAHD-family monolignol acyltransferase, mediates lignin acylation in poplar. Plant Physiol..

[37] Ullah C., Chen Y.H., Ortega M.A., Tsai C.J. (2023). The diversity of salicylic acid biosynthesis and defense signaling in plants: Knowledge gaps and future opportunities. Curr. Opin. Plant Biol..

[38] Kriegshauser L., Knosp S., Grienenberger E., Tatsumi K., Gütle D.D., Sørensen I., Herrgott L., Zumsteg J., Rose J.K.C., Reski R. (2021). Function of the HYDROXYCINNAMOYL-CoA:SHIKIMATE HYDROXYCINNAMOYL TRANSFERASE is evolutionarily conserved in embryophytes. Plant Cell.

[39] Wang P.P., Liu H., Gao S., Cheng A.X. (2017). Functional Characterization of a Hydroxyacid/Alcohol Hydroxycinnamoyl Transferase Produced by the Liverwort *Marchantia emarginata*. Molecules.

[40] Abdullah, Faraji S., Heidari P., Poczai P. (2021). The BAHD Gene Family in Cacao (*Theobroma cacao*, Malvaceae): Genome-Wide Identification and Expression Analysis. Front. Ecol. Evol..

[41] Liu L., Xu H., Zhang W., Xing J., Zhu M., Zhang Y., Wang Y. (2023). Genome-Wide Analysis of the BAHD Family in Welsh Onion and CER2-LIKEs Involved in Wax Metabolism. Genes.

[42] Walker K., Schoendorf A., Croteau R. (2000). Molecular cloning of a taxa-4(20),11(12)-dien-5alpha-ol-O-acetyl transferase cDNA from Taxus and functional expression in *Escherichia coli*. Arch. Biochem. Biophys..

[43] Moore R.C., Purugganan M.D. (2003). The early stages of duplicate gene evolution. Biol. Sci..

[44] Cannon S.B., Mitra A., Baumgarten A., Young N.D., May G. (2004). The roles of segmental and tandem gene duplication in the evolution of large gene families in *Arabidopsis thaliana*. BMC Plant Biol..

[45] Zhu Y., Wu N., Song W., Yin G., Qin Y., Yan Y., Hu Y. (2014). Soybean (Glycine max) expansin gene superfamily origins: Segmental and tandem duplication events followed by divergent selection among subfamilies. BMC Plant Biol..

[46] Ramamoorthy R., Jiang S.Y., Kumar N., Venkatesh P.N., Ramachandran S. (2008). A comprehensive transcriptional profiling of the WRKY gene family in rice under various abiotic and phytohormone treatments. Plant Cell Physiol..

[47] Hanada K., Zou C., Lehti-Shiu M.D., Shinozaki K., Shiu S.H. (2008). Importance of lineage-specific expansion of plant tandem duplicates in the adaptive response to environmental stimuli. Plant Physiol..

[48] Tuominen L.K., Johnson V.E., Tsai C.J. (2011). Differential phylogenetic expansions in BAHD acyltransferases across five angiosperm taxa and evidence of divergent expression among *Populus paralogues*. BMC Genom..

[49] Souleyre E.J., Greenwood D.R., Friel E.N., Karunairetnam S., Newcomb R.D. (2005). An alcohol acyl transferase from apple (cv. Royal Gala), MpAAT1, produces esters involved in apple fruit flavor. FEBS J..

[50] Li D., Xu Y., Xu G., Gu L., Li D., Shu H. (2006). Molecular cloning and expression of a gene encoding alcohol acyltransferase (MdAAT2) from apple (cv. Golden Delicious). Phytochemistry.

[51] El-Sharkawy I., Manríquez D., Flores F.B., Regad F., Bouzayen M., Latché A., Pech J.C. (2005). Functional characterization of a melon alcohol acyl-transferase gene family involved in the biosynthesis of ester volatiles. Identification of the crucial role of a threonine residue for enzyme activity *. Plant Mol. Biol..

[52] Boatright J., Negre F., Chen X., Kish C.M., Wood B., Peel G., Orlova I., Gang D., Rhodes D., Dudareva N. (2004). Understanding in vivo benzenoid metabolism in petunia petal tissue. Plant Physiol..

[53] Xu Y., Tie W., Yan Y., Xu B., Liu J., Li M., Yang J., Zeng J., Hu W., Jin Z. (2021). Identification and expression of the BAHD family during development, ripening, and stress response in banana. Mol. Biol. Rep..

[54] Suzuki H., Nakayama T., Nishino T. (2003). Proposed mechanism and functional amino acid residues of malonyl-CoA:anthocyanin 5-O-glucoside-6‴-O-malonyltransferase from flowers of Salvia splendens, a member of the versatile plant acyltransferase family. Biochemistry.

[55] Bayer A., Ma X., Stöckigt J. (2004). Acetyltransfer in natural product biosynthesis--functional cloning and molecular analysis of vinorine synthase. Bioorg. Med. Chem..

[56] Unno H., Ichimaida F., Suzuki H., Takahashi S., Tanaka Y., Saito A., Nishino T., Kusunoki M., Nakayama T. (2007). Structural and mutational studies of anthocyanin malonyltransferases establish the features of BAHD enzyme catalysis. J. Biol. Chem..

[57] Tamura K., Dudley J., Nei M., Kumar S. (2007). MEGA4: Molecular Evolutionary Genetics Analysis (MEGA) software version 4.0. Mol. Biol. Evol..

[58] Ma X., Koepke J., Panjikar S., Fritzsch G., Stöckigt J. (2005). Crystal structure of vinorine synthase, the first representative of the BAHD superfamily *. J. Biol. Chem..

[59] Zhang W., Li J., Dong Y., Huang Y., Qi Y., Bai H., Li H., Shi L. (2024). Genome-wide identification and expression of BAHD acyltransferase gene family shed novel insights into the regulation of linalyl acetate and lavandulyl acetate in lavender. J. Plant Physiol..

[60] Zhuang W., Shu X., Lu X., Wang T., Zhang F., Wang N., Wang Z. (2021). Genome-wide analysis and expression profiles of PdeMYB transcription factors in colored-leaf poplar (*Populus deltoids*). BMC Plant Biol..

[61] Murayama K., Kato-Murayama M., Sato T., Hosaka T., Ishiguro K., Mizuno T., Kitao K., Honma T., Yokoyama S., Tanaka Y. (2021). Anthocyanin 5,3′-aromatic acyltransferase from *Gentiana triflora*, a structural insight into biosynthesis of a blue anthocyanin. Phytochemistry.

[62] Zhang J.F., Gong S., Guo Z.G. (2010). Effects of different elicitors on 10-deacetylbaccatin III-10-O-acetyltransferase activity and cytochrome P450 monooxygenase content in suspension cultures of *Taxus cuspidata* cells. Cell Biol. Int. Rep..

[63] Baebler S., Camloh M., Kovac M., Ravnikar M., Zel J. (2002). Jasmonic acid stimulates taxane production in cell suspension culture of yew (Taxus x media). Planta Med..

[64] Hao D.C., Ge G., Xiao P., Zhang Y., Yang L. (2011). The first insight into the tissue specific taxus transcriptome via Illumina second generation sequencing. PLoS ONE.

[65] Ashihara H., Ludwig I.A., Crozier A. (2020). Biosynthesis of Purine Alkaloids. Plant Nucleotide Metabolism—Biosynthesis, Degradation, and Alkaloid Formation.

[66] Nevarez D.M., Mengistu Y.A., Nawarathne I.N., Walker K.D. (2009). An N-aroyltransferase of the BAHD superfamily has broad aroyl CoA specificity in vitro with analogues of N-dearoylpaclitaxel. J. Am. Chem. Soc..

[67] Finn R.D., Clements J., Eddy S.R. (2011). HMMER web server: Interactive sequence similarity searching. Nucleic Acids Res..

[68] Letunic I., Khedkar S., Bork P. (2021). SMART: Recent updates, new developments and status in 2020. Nucleic Acids Res..

[69] Lu S., Wang J., Chitsaz F., Derbyshire M.K., Geer R.C., Gonzales N.R., Gwadz M., Hurwitz D.I., Marchler G.H., Song J.S. (2020). CDD/SPARCLE: The conserved domain database in 2020. Nucleic Acids Res..

[70] Horton P., Park K.J., Obayashi T., Fujita N., Harada H., Adams-Collier C.J., Nakai K. (2007). WoLF PSORT: Protein localization predictor. Nucleic Acids Res..

[71] Tamura K., Stecher G., Kumar S. (2021). MEGA11: Molecular Evolutionary Genetics Analysis Version 11. Mol. Biol. Evol..

[72] Letunic I., Bork P. (2021). Interactive Tree Of Life (iTOL) v5: An online tool for phylogenetic tree display and annotation. Nucleic Acids Res..

[73] Viner C., Ishak C.A., Johnson J., Walker N.J., Shi H., Sjöberg-Herrera M.K., Shen S.Y., Lardo S.M., Adams D.J., Ferguson-Smith A.C. (2024). Modeling methyl-sensitive transcription factor motifs with an expanded epigenetic alphabet. Genome Biol..

[74] Chen C., Chen H., Zhang Y., Thomas H.R., Frank M.H., He Y., Xia R. (2020). TBtools: An Integrative Toolkit Developed for Interactive Analyses of Big Biological Data. Mol. Plant.

[75] Chao J.T., Kong Y.Z., Wang Q., Sun Y.H., Gong D.P., Lv J., Liu G.S. (2015). MapGene2Chrom, a tool to draw gene physical map based on Perl and SVG languages. Yi Chuan Hered..

[76] Wang Y., Tang H., Debarry J.D., Tan X., Li J., Wang X., Lee T.H., Jin H., Marler B., Guo H. (2012). MCScanX: A toolkit for detection and evolutionary analysis of gene synteny and collinearity. Nucleic Acids Res..

[77] Lescot M., Déhais P., Thijs G., Marchal K., Moreau Y., Van de Peer Y., Rouzé P., Rombauts S. (2002). PlantCARE, a database of plant cis-acting regulatory elements and a portal to tools for in silico analysis of promoter sequences. Nucleic Acids Res..

[78] Livak K.J., Schmittgen T.D. (2001). Analysis of relative gene expression data using real-time quantitative PCR and the 2(-Delta Delta C(T)) Method. Methods.

[79] Kęska K., Szcześniak M.W., Makałowska I., Czernicka M. (2021). Long-Term Waterlogging as Factor Contributing to Hypoxia Stress Tolerance Enhancement in Cucumber: Comparative Transcriptome Analysis of Waterlogging Sensitive and Tolerant Accessions. Genes.

[80] Li L., Chen Y., Ma Y., Wang Z., Wang T., Xie Y. (2021). Optimization of Taxol Extraction Process Using Response Surface Methodology and Investigation of Temporal and Spatial Distribution of Taxol in *Taxus mairei*. Molecules.

